# Comprehensive dosimetric comparison of halcyon 3.0 versus truebeam for breast cancer volumetric modulated arc therapy plans

**DOI:** 10.3389/fonc.2025.1630702

**Published:** 2025-08-20

**Authors:** Anyan Gu, Mengyuan Wang, Zhenyu Pan, Ran Tang, Yaotao Li, Yu Wu, Guozi Yang, Xingru Sun

**Affiliations:** ^1^ Department of Radiation Oncology, The Affiliated Huizhou Hospital, Guangzhou Medical University, Huizhou, China; ^2^ Radiation Therapy Zone, Central Hospital of Guangdong Provincial Nongken, Zhanjiang, China

**Keywords:** halcyon, truebeam, breast cancer, volumetric modulated arc therapy, radiotherapy plan

## Abstract

**Background:**

Breast cancer is the foremost malignancy threatening female health. This study aimed to compare the dosimetric performance of Halcyon 3.0 and TrueBeam in Volumetric Modulated Arc Therapy (VMAT) planning for breast cancer.

**Methods:**

Thirty post-operative breast cancer patients were included. VMAT plans for Halcyon 3.0 and TrueBeam were generated using 6MV FFF beams in Eclipse 16.1 with identical optimizing objectives. Organs At Risk (OARs) and target metrics were evaluated. Statistical analysis employed paired t-tests or Wilcoxon signed-rank tests (α=0.05).

**Results:**

Halcyon significantly reduced the mean dose of heart (Δ = −112.5 cGy, P< 0.001), left anterior descending artery mean dose (Δ = −151.3 cGy, P< 0.001), and spinal cord maximum dose (Δ = −88.2 cGy, P = 0.011). Low-dose exposure improved, with heart V5 (volume receiving ≥500 cGy) reduced from 22.0% to 12.6% (P< 0.001) and ipsilateral lung V5 reduced from 59.4% to 45.8% (P< 0.001) between Halcyon and TrueBeam. Halcyon lowered the absolute volume received 500 cGy by 487 cm³ (P< 0.001). Halcyon had the better target homogeneity (homogeneity index: Δ = -0.01, P< 0.001), while conformity remained comparable (P > 0.05). Despite having higher monitor units (Δ = 87.5, P< 0.001), Halcyon maintained delivery efficiency through faster leaf motion speed and gantry rotation velocity.

**Conclusions:**

Compared with TrueBeam, Halcyon achieved reduction in low-dose exposure while maintaining target coverage in breast cancer VMAT planning, thereby lowering radiation doses to OARs (e.g., heart and lung V5). This benefit is likely attributed to Halcyon’s unique staggered dual-layer MLC design.

## 1 Introduction

Breast cancer is the foremost malignancy threatening female health, accounting for 11.6% of global cancer diagnoses in 2022 (1). Its rising incidence among younger individuals heightens the urgent need to develop strategies aimed at prolonging patient survival (2). Radiotherapy, a cornerstone of multimodal therapeutic strategies, effectively reduces locoregional recurrence rates, extends survival, and lowers breast cancer-related mortality (3). However, given the emphasis on long-term survivorship in breast cancer patients, stricter dose constraints for organs at risk (OARs) are required compared to other cancers to minimize radiation-induced sequelae (4). Among long-term survivors, cardiovascular complications are the predominant non-oncologic cause of mortality, with major coronary events increasing linearly with mean cardiac dose (7.4% excess risk per 1 Gy increment) (5, 6). Other complications include a 1.3% incidence of grade 2 radiation pneumonitis from comprehensive nodal irradiation and thyroid dysfunction from supraclavicular field irradiation (7). Additionally, excessive radiation exposure to the contralateral breast may lead to secondary malignancies (8). These findings highlight the imperative for meticulous minimization of OAR doses during treatment planning to preserve survivorship quality.

Post-mastectomy radiotherapy (PMRT) typically targets the chest wall, with optional inclusion of nodal regions (supraclavicular, axillary), and internal mammary nodes (9). Breast-conserving radiotherapy primarily targets the whole breast, though it may also involve nodal regions and internal mammary nodes when necessary. The plan optimization of these therapies faces several challenges, including the irregular surface contours, the pronounced curvature of the planning target volumes (PTV), cutaneous proximity, and critical organ adjacency. Currently, the main clinical treatment modalities include Three-dimensional Conformal Radiotherapy (3D-CRT), Intensity-modulated Radiation Therapy (IMRT), and Volumetric Modulated Arc Therapy (VMAT). VMAT, an evolution of IMRT, enhances conformity to highly curved target geometries through synchronized modulation of gantry rotation velocity, multi-leaf collimator (MLC) positioning, and dose rate variability, while simultaneously reducing delivery times compared to conventional IMRT (10, 11). Superior PTV dose homogeneity in left-sided breast cancer VMAT planning has been demonstrated by Zhao et al. (12) and Narudom et al. (13). Additionally, the OAR-sparing advantages of VMAT have been reported by Evgenia et al. (14) and Liu et al. (15). Consequently, VMAT-based techniques were employed for all cases in this study.

Recently, Varian introduced the Halcyon 3.0 ring-gantry treatment delivery system, featuring an “armless” ring-shaped design and dual-layer staggered 1 cm-width MLC (achieving an effective resolution of 5 mm at isocenter). And they assert that the Halcyon system achieves the transmission of 0.01% (maybe reducing low-dose exposure). This study compared the dosimetric differences between the Halcyon 3.0 and TrueBeam systems in 30 post-operative breast cancer patients, particularly in low-dose exposure regions. The purpose of this research is to provide a clinical reference for optimizing breast cancer radiotherapy plans.

## 2 Materials and methods

### 2.1 Case selection

This retrospective study enrolled 30 female breast cancer patients treated with adjuvant radiotherapy following modified radical mastectomy (MRM) or breast-conserving surgery (BCS) at our institution between July 2024 and February 2025. The cohort comprised 14 left-sided MRM cases, 10 right-sided MRM cases, 3 left-sided BCS cases, and 3 right-sided BCS cases. Patients aged 30–76 years (mean 52.37 years) were diagnosed with early-stage breast cancer (T1-T2), graded 1–3, without distant metastasis. Demographic and clinical characteristics were summarized in **Table 1**. As this was a retrospective study, informed consent was obtained from all participating patients.

**Table 1:** Patients’ characteristics and planning target volume details.

Characteristic	Frequency
Age (year)	<30|52.37 ± 10.9|76>
≥55	37%
<55	63%
Diagnosis	
Left-Sided Breast Cancer	17
Right-Sided Breast Cancer	13
Surgical procedure	
Post-mastectomy radiotherapy (PMRT)	24
Post-lumpectomy radiotherapy (PIRT)	6
PTV	
PTVcw+PTVsc	5
PTVcw+PTVsc+PTVax	3
PTVcw+PTVsc+PTVim	6
PTVcw+PTVsc+PTVax+PTVim	7
PTVcw+PTVsc+PGTVtb	1
PTVcw+PTVsc+PTVax+PGTVtb	1
PTVcw+PTVsc+PTVax+PTVim+PGTVtb	1
PTVb+PTVsc+PTVboost	2
PTVb+PTVsc+PTVax+PTVboost	2
PTVb+PTVsc+PTVim+PTVboost	2

### 2.2 CT simulation

All patients were immobilized using the latest CIVCO breast board system combined with vacuum cushions in a head-first supine position. The upper limbs were elevated with hands gripping a positioning rod, and the head was rotated toward the contralateral side to fully expose the neck and supraclavicular regions. For post-MRM cases, two CT scans were acquired under identical positioning conditions: one with a 0.5 cm bolus and one without. The treatment plan for the first 15 fractions was optimized using the CT scan with bolus, and radiation delivery was performed with the bolus applied. For the subsequent 10 fractions, the treatment plan was re-optimized using the CT scan without bolus, and treatments were delivered without the bolus.

CT scanning was performed using a Philips Brilliance Big Bore scanner (Philips, Eindhoven, Netherlands), with the scanning range extending from the mandible to 1 cm below the diaphragm to ensure complete inclusion of potential OARs for dosimetric optimization and plan evaluation. Axial slices were acquired at 3–5 mm thickness.

### 2.3 Contouring of CTV and OARs

**Figure 1:**
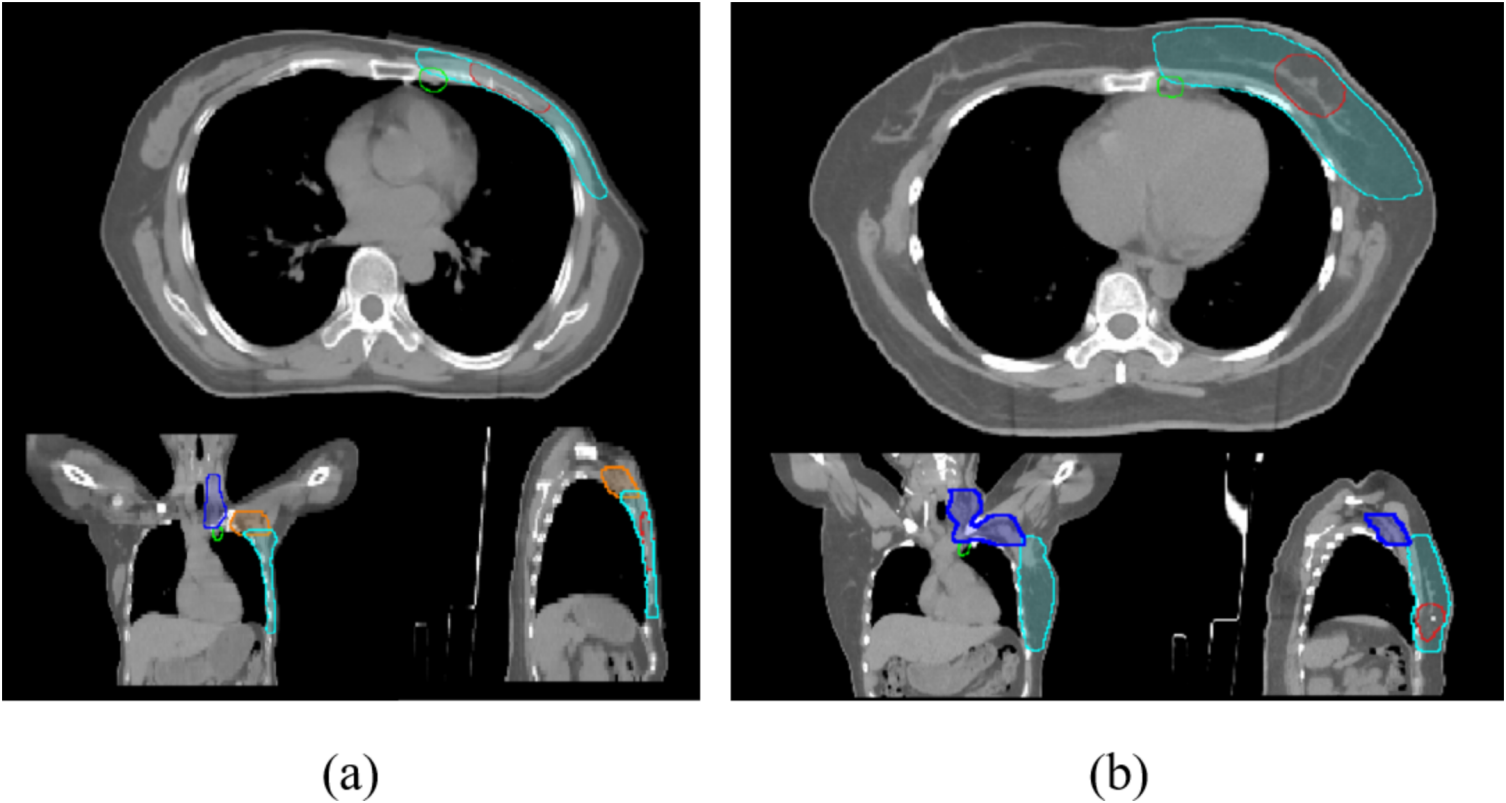
**(a)** Cross-sectional views of planning target volumes (PTVs margined from the CTVs) for post-mastectomy patients; **(b)**: PTVs for breast-conserving surgery patients. Light blue: PTVcw/PTVb; dark blue: PTVsc; orange: PTVax; green: PTVim; red: PGTVtb/PTVboost.

Clinical target volumes (CTVs) were delineated according to the surgical approach. For post-MRM cases, CTVs included the ipsilateral chest wall (CTVcw), supraclavicular region (CTVsc), axillary lymphatic drainage area (CTVax) and internal mammary nodes (CTVim), and sometimes the gross tumor volume (GTVtb) (**Figure 1a**). For post-BCS cases, CTVs incorporated the ipsilateral whole breast (CTVb), tumor bed (CTVboost), CTVsc, CTVim and CTVax (**Figure 1b**). OAR contours were initially generated using the Varian AI-based auto-segmentation software (RTMindProcess) and were subsequently reviewed, modified, and approved by experienced radiation oncologists. Target volumes were manually delineated concurrently.

### 2.4 Linac (linear accelerator) characteristics and treatment planning

The Halcyon 3.0 system features a double-layer staggered MLC design with 1 cm-width leaves, comprising 29 pairs proximal to the source and 28 pairs distal to the source, achieving an effective isocentric resolution of 5 mm (due to the double-layer staggered MLC design). The maximum leaf travel is 28 cm, with a maximum field size of 28×28 cm². The maximum dose rate is capped at a maximum of 800 MU/min. In contrast, the TrueBeam system incorporates dual backup jaws and millennium 120 MLC leaves in the X-direction, providing an isocentric resolution of 5 mm at the center and 10 mm in the peripheral regions. The maximum leaf travel is 15 cm, with a field size limit of 40×40 cm², and a dose rate of a maximum of 1400 MU/min.

All 30 patients were treated clinically using the Halcyon 3.0 system. For comparative analysis, TrueBeam-based plans were retrospectively generated for the same cohort. Treatment planning was performed on the Eclipse 16.1 treatment planning system (TPS, Varian Medical Systems) using 6 MV flattening filter-free (FFF) photon beams. Maximum dose rates were 800 MU/min for Halcyon and 1400 MU/min for TrueBeam. The identical optimization engine (Photon Optimizer) and the Acuros XB dose calculation algorithm (2.5 mm grid resolution) were utilized for both platforms. TrueBeam planning parameters, including dose constraints and NTO, were rigorously matched to the Halcyon setup.

**Figure 2:**
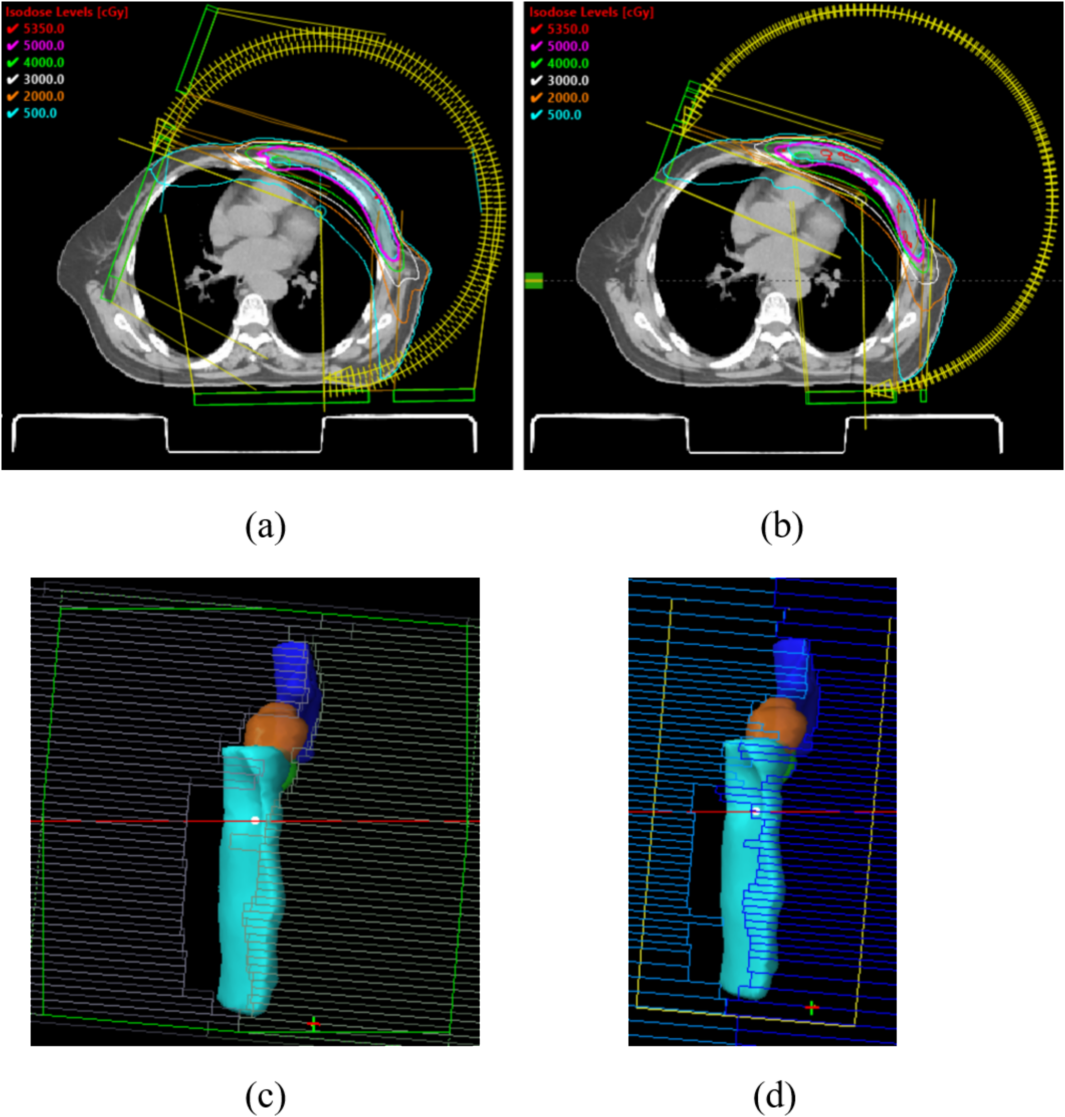
**(a, b)** The arc arrangements and dose distributions for Halcyon **(a)** and TrueBeam **(b)**; **(c, d)** expands field coverage beyond the chest wall under the influence of the virtual bolus in Halcyon **(c)** and TrueBeam **(d)**.

All plans employed partial-arc VMAT techniques. For left-sided breast cases, arcs spanned clockwise from 290°/300° to 179°/160°, while right-sided cases utilized clockwise arcs from 181°/200° to 60°/70°. Each plan included 3–4 arcs (depending on optimization complexity). Collimator angles were set at 0°, 5°, 10°, 350°, or 355°. **Figures 2a, b** illustrates the arc arrangements and dose distributions for Halcyon (a) and TrueBeam (b). Given that the breast is located in the thoracic region, respiratory motion can displace the target volume beyond the radiation field during treatment. To mitigate this, a 2 cm-wide virtual bolus was incorporated into the planning process. This approach has been validated by multiple studies and effectively expands field coverage beyond the chest wall in VMAT breast plans (**Figures 2c, d**) (16–18). In this study, we compared the mean dose distribution within the virtual bolus-expanded fields to evaluate potential differences in the performance of the Halcyon and TrueBeam systems under this virtual bolus configuration.

Prescription doses adhered to guideline-recommended conventional fractionation: 2 Gy per fraction over 25 daily fractions for PTVs (50 Gy/25F), while PTVboost/PGTVtb received 2.4 Gy per fraction across the same 25-fraction regimen (60 Gy/25F). The planning requirements mandated that 95% of the PTV volume receive ≥50 Gy (PTVs) and ≥60 Gy (PTVboost/PGTVtb). The dosimetric evaluation focused on OAR sparing for the heart (Dmean [mean dose], V20 [volume receiving ≥20 Gy], V5 [volume receiving ≥5 Gy]), LAD (V20 and Dmean, analyzed for left-sided cases only), contralateral breast (V20, V5, Dmean), ipsilateral/contralateral lungs (V20, V5, Dmean), liver (V5, Dmean), stomach (V5, Dmean, left-sided cases only), spinal cord and spinal cord PRV (Dmax), and thyroid (Dmean). Furthermore, the absolute volumes receiving 500 cGy (V5) have been compared between the two systems (to further explore the difference in lower radiation leakage). Clinical objectives were established in accordance with the International Commission on Radiation Units (ICRU) and Radiation Therapy Oncology Group (RTOG) guidelines, as detailed in **Table 2**.

**Table 2:** Clinical objectives.

Structure	Clinical objectives	Acceptable values
PTVcw/PTVb	V110%<0.5%	V110%<1%
	V107%<8%	V107%<10%
Heart	Dmean<5Gy	Dmean<7Gy
	V20<5%	V20<8%
	V5<40%	V5<50%
LAD	V40<20%	V40<25%
Contralateral Lung	V5<20%	V5<25%
	Dmean<5Gy	Dmean<6Gy
Ipsilateral Lung	V20<25%	V20<28%
	V5<55%	V5<60%
	Dmean<13Gy	Dmean<15Gy
Lungs	V20<15%	V20<20%
	V5<45%	V5<55%
	Dmean<10Gy	Dmean<12Gy
Contralateral Breast	Dmean<5Gy	Dmean<7Gy
Spinal cord	Dmax<30Gy	Dmax<35Gy
Spinal cord PRV(+5mm)	Dmax<40Gy	Dmax<45Gy
Thyroid	Dmean<21Gy	Dmean<23Gy
Liver	V5<10%	V5<20%
Stomach	V5<10%	V5<20%

Vx, Volume receiving ≥X Gy dose. Specifically, this study focuses on hotspots in the chest wall (PTVcw) and whole breast (PTVb), V107%, Volume receiving ≥107% of the prescription dose; V110%, Volume receiving ≥110% of the prescription dose; Dmean ,Mean dose; Dmax, Maximum dose.

For PTV evaluation, HI (from ICRU-83) Equation 1 and Paddick CI (Equation 2) (19) were analyzed as follows:


(1) $$\text{HI}=\frac{\text{D}2\%-\text{D}98\%}{\text{D}50\%}$$

where D2%, D98% and D50% are the doses to 2%, 98% and 50% volume of PTV (applied to PTVcw and PTVb).


(2) $$\text{CI}=\frac{{\left(TV\text{PIV}\right)}^{2}}{TV•PIV}$$

where TVPIV is the target volume covered by the prescription isodose, TV is the target volume, and PIV is the prescription isodose volume (applied to all PTVs). This study also evaluated the PTVcw/PTVb volumes receiving 107% and 110% of the prescription dose.

Lastly, Plan verification was conducted using Varian’s Portal Dosimetry, with gamma passing rates evaluated using 3%/2 mm criteria (γ[3%, 2 mm]< 1). A passing rate of ≥ 95% was enforced for clinical acceptance.

### 2.5 Statistical analysis

All statistical analyses were conducted using IBM SPSS Statistics (Version 26.0, Armonk, NY). The normality of continuous variables was assessed through the Shapiro-Wilk test. Paired comparisons between Halcyon and TrueBeam plans were performed using the paired t-test for normally distributed data. A two-tailed significance level of α = 0.05 was predefined for all inferential analyses.

## 3 Results

As shown in **Table 1**, 63% of the patients were under 55 years old, which aligns with the previously noted trend of increasing breast cancer incidence in younger populations. The inclusion of diverse target volumes (post-mastectomy and breast-conserving cases) enhanced the generalizability of our findings.

### 3.1 Dosimetric comparison of OARs

**Figure 3:**
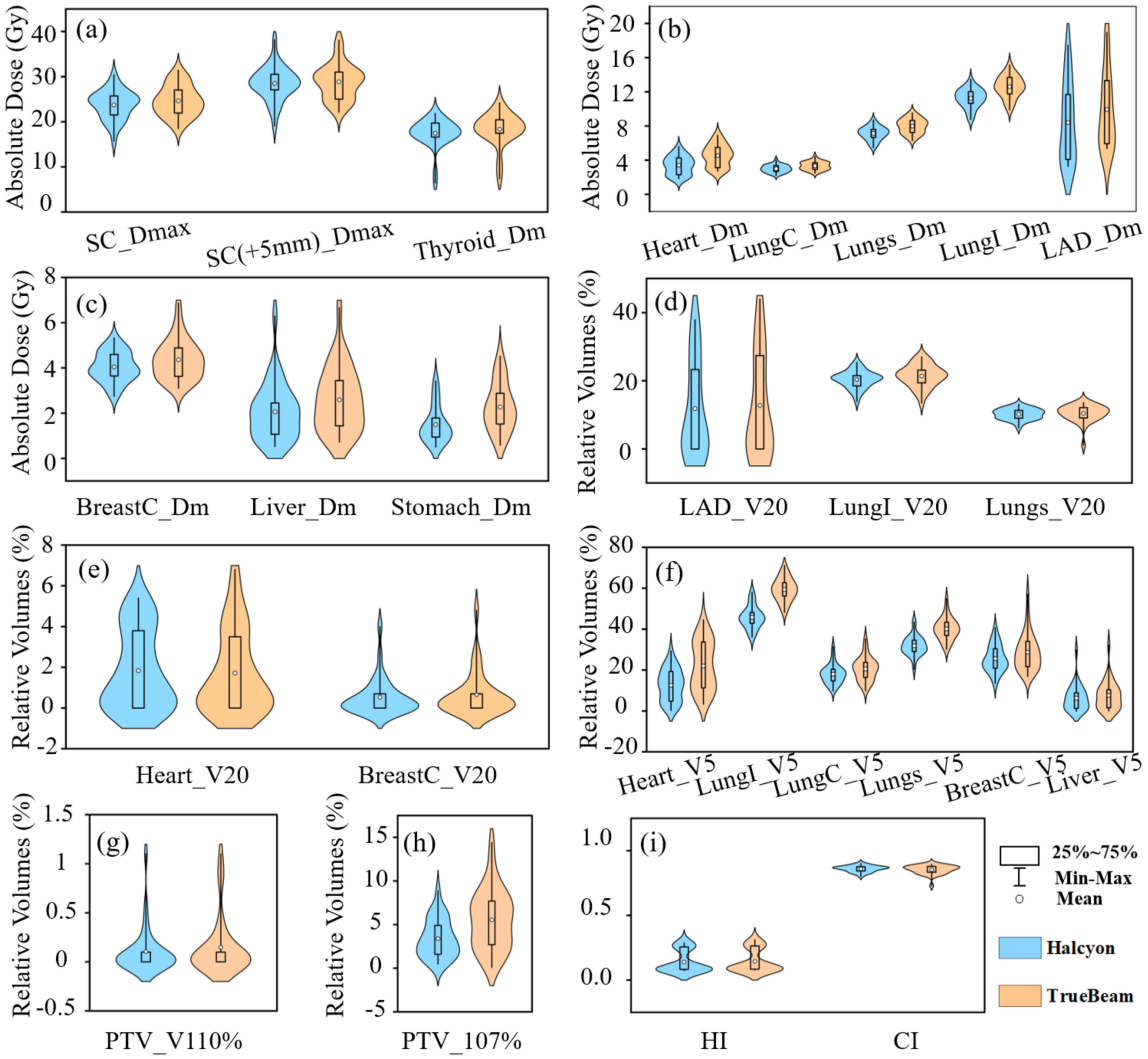
Violin plot showing. **(a-c)** absolute dose metrics, LungI (ipsilateral lung), LungC (contralateral lung), and BreastC (contralateral breast), SC (spinal cord), Dm(Dmean); **(d-f)** relative volume metrics; **(g-i)** Parameters about PTV evaluation.

Compared with TrueBeam, Halcyon demonstrated significantly reduced radiation doses, especially in low-dose regions (e.g., V5), to critical OARs. A significant mean difference (MD) of -112 cGy (P< 0.001) was observed for the heart Dmean between treatment plans optimized on Halcyon versus TrueBeam platforms, representing a 24.8% reduction using Halcyon. Similarly, Halcyon reduced the heart V5 (Δ = -9.4%, P< 0.001), although no significant difference was observed in V20 (Δ = 0.1%, P > 0.05). The Dmean of LAD decreased by 152cGy in Halcyon (P< 0.001). The MD of Ipsilateral lung V20 and V5 were -1.0% (P< 0.001) and -13.6% (P< 0.001), respectively, while contralateral breast V5 decreased by 3.2% (P = 0.002). Notably, Halcyon also achieved lower spinal cord Dmax (Δ = -88.2 cGy, P = 0.01) and thyroid Dmean (Δ = -88 cGy, P< 0.001). **Figures 3a–c** illustrates the absolute dose distributions across OARs, highlighting Halcyon’s tighter dose distributions (IQR [interquartile range]: 280–410 cGy vs. 380–520 cGy for heart Dmean) and median reductions exceeding 100cGy for Dmean of heart and LAD. **Figures 3d–f** demonstrates that the V5 for all OARs in Halcyon plans was significantly lower compared to TrueBeam plans. **Figures 3g–i** show the comparison results of PTV-related parameters.

### 3.2 Target coverage and treatment efficiency

No significant differences in CI were observed (P > 0.05), but Halcyon achieved a better HI (Δ = -0.01, P< 0.001) (**Table 3**). Halcyon significantly reduced hotspot volumes (Δ = -2.1%, P< 0.001), although differences in V110% were non-significant (P > 0.05). As shown in **Figure 3c**, V107% values for Halcyon plans were clustered within 1–5%, whereas TrueBeam plans exhibited a broader distribution (3–9%). Despite higher monitor units (MU) with Halcyon (Δ = 87.5MU, P< 0.001), its faster leaf motion speed and gantry rotation velocity resulted in comparable overall treatment time to TrueBeam.

**Table 3:** Dosimetric comparison of PTV and OARs between Halcyon and TrueBeam.

Structure	Parameters	Halcyon	TrueBeam	P
PTV	V110%(%)	0.1 ± 0.3	0.1 ± 0.3	P(t)=0.165
	V107%(%)	3.4 ± 2.0	5.5 ± 3.6	**P(t)<0.001**
	HI	0.14 ± 0.09	0.15 ± 0.09	**P(t)<0.001**
	CI	0.86 ± 0.02	0.85 ± 0.04	P(w)=0.156
Heart	V20(%)	1.8 ± 1.9	1.7 ± 2.0	P(t)=0.371
	V5(%)	12.6 ± 7.9	22.0 ± 12.1	**P(t)<0.001**
	Dmean(cGy)	340.7 ± 108.0	453.7 ± 127.2	**P(t)<0.001**
LAD	V20(%)	11.9 ± 14.1	12.8 ± 15.4	P(w)=0.563
	Dmean(cGy)	841.8 ± 472.0	993.2 ± 454.6	**P(w)<0.001**
Ipsilateral Lung	V20(%)	20.4 ± 2.6	21.4 ± 3.3	**P(t)<0.001**
	V5(%)	45.8 ± 5.6	59.4 ± 5.6	**P(t)<0.001**
	Dmean(cGy)	1125.0 ± 113.3	1260.4 ± 128.7	**P(t)<0.001**
Contralateral Lung	V5(%)	18.0 ± 4.8	20.6 ± 5.9	**P(t)=0.008**
	Dmean(cGy)	300.8 ± 44.1	333.9 ± 48.3	**P(t)<0.001**
Lungs	V20(%)	10.2 ± 1.7	10.4 ± 2.6	P(t)=0.636
	V5(%)	31.9 ± 4.9	40.1 ± 5.5	**P(t)<0.001**
	Dmean(cGy)	714.6 ± 78.9	798.8 ± 87.1	**P(t)<0.001**
Contralateral Breast	V20(%)	0.5 ± 0.9	0.7 ± 1.1	P(t)=0.120
	V5(%)	25.6 ± 6.6	28.8 ± 9.2	**P(t)=0.009**
	Dmean(cGy)	404.8 ± 66.4	436.9 ± 89.2	**P(t)<0.001**
Spinal cord	Dmax(cGy)	2376.6 ± 323.3	2464.8 ± 338.8	**P(w)=0.011**
Spinal cord PRV(+5mm)	Dmax(cGy)	2849.2 ± 372.7	2888.4 ± 393.4	P(w)=0.239
Thyroid	Dmean(cGy)	1748.4 ± 316.2	1836.4 ± 363.3	**P(t)<0.001**
Liver	V5(%)	6.3 ± 6.5	7.6 ± 7.3	**P(t)=0.011**
	Dmean(cGy)	206.4 ± 127.3	259.8 ± 143.4	**P(w)<0.001**
Stomach	V5(%)	0.3 ± 0.5	1.0 ± 1.7	P(w)=0.095
	Dmean(cGy)	149.5 ± 81.5	227.8 ± 108.5	**P(w)<0.001**

Using paired t-test or the Wilcoxon signed-rank test, with statistical significance defined as P ≤ 0.05. Where P(t) denotes the p-value from the t-test, and P(w) represents the p-value from the Wilcoxon signed-rank test (values with P<0.05 are shown in bold).

**Figure 4:**
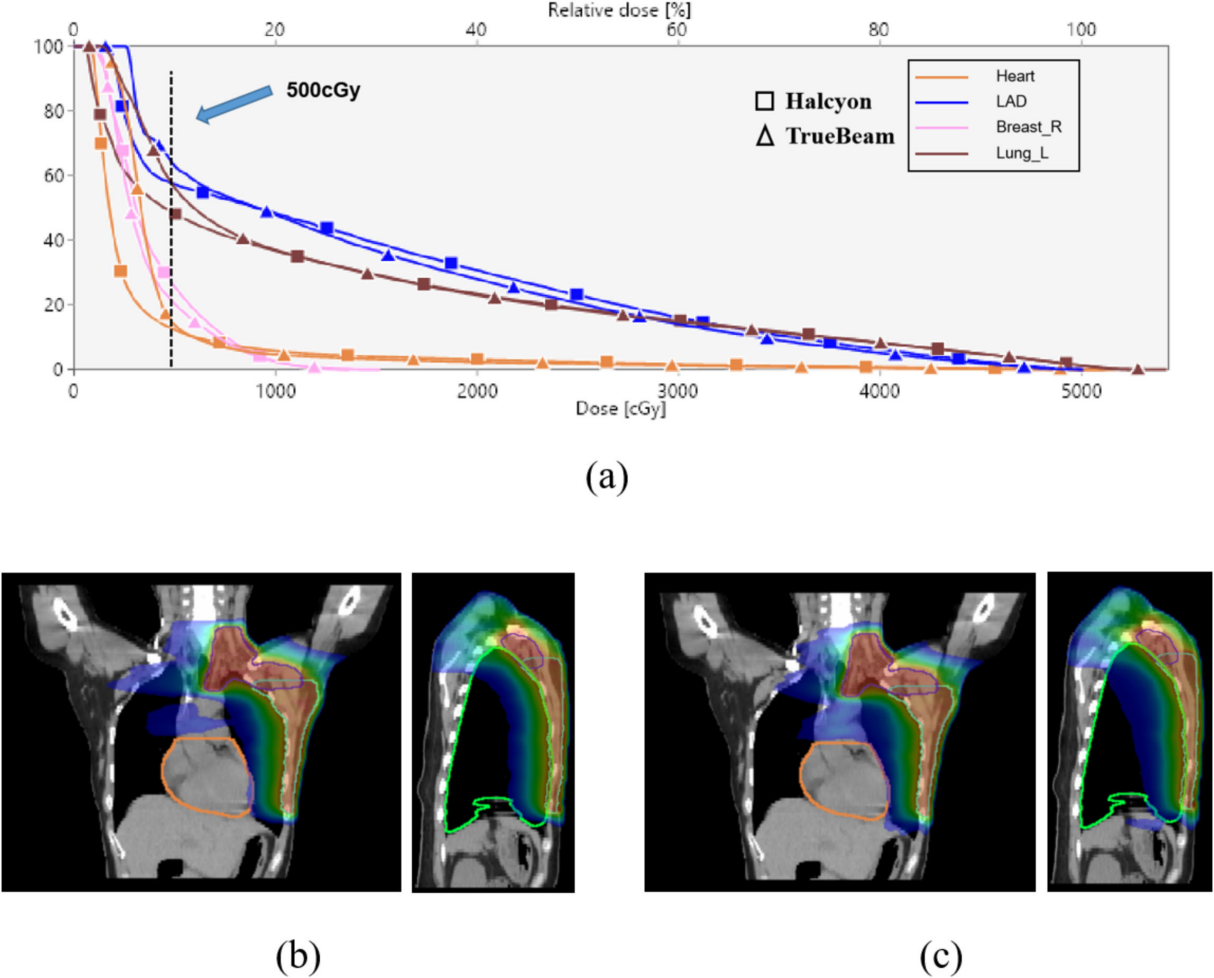
DVH and dose distribution analysis. **(a)** DVH curves for critical OARs (heart, LAD, ipsilateral lung, and contralateral breast) for Halcyon and TrueBeam; **(b, c)** Coronal and sagittal dose distributions for Halcyon **(b)** and TrueBeam **(c)**, heart is orange, ipsilateral lung is green, with the outermost blue isodose line representing 500 cGy.

**Figure 4** demonstrates dose distributions in a representative case, where Halcyon’s dose-volume histograms (DVHs) for the heart and lungs shifted leftward in low-dose regions (< 500 cGy) (**Figure 4a**), and axial dose clouds confirmed enhanced cardiac sparing (**Figures 4b, c**).

### 3.3 Additional characteristics and plan dose verification

**Figure 5:**
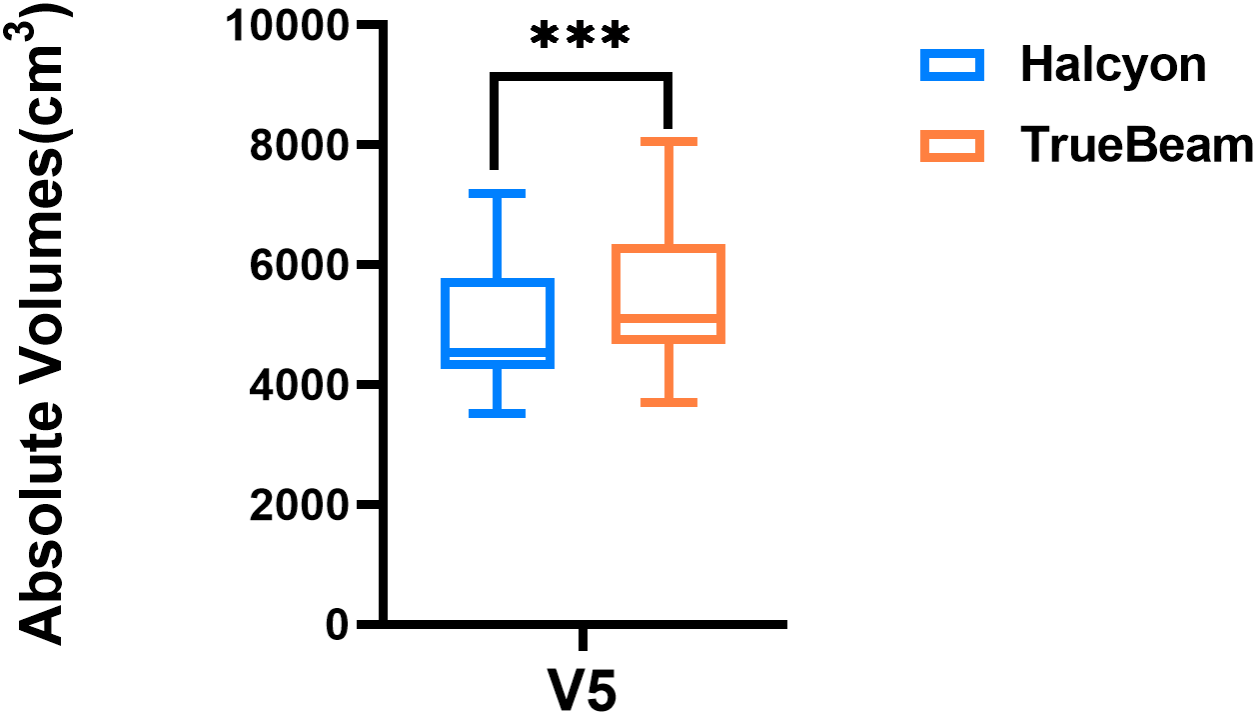
Box plot comparing absolute volumes receiving 500 cGy (V5) between Halcyon and TrueBeam treatment plans. *** denotes statistical significance at P<0.0001.

**Figure 6:**
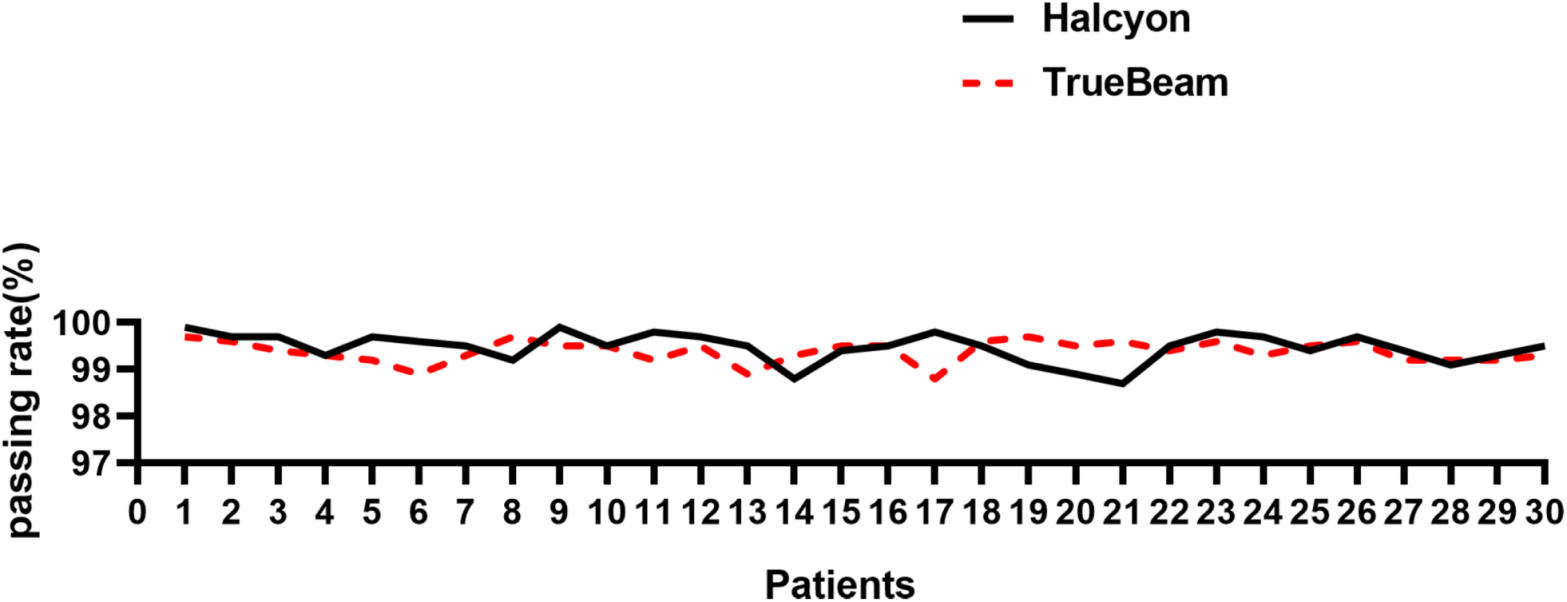
Comparison of γ [3%, 2 mm] passing rates between Halcyon and TrueBeam for 30 breast cancer plans.

**Figure 5** shows a statistically significant superiority of Halcyon compared to TrueBeam in V5, with an MD of 487.2 cm^3^ (P< 0.001). In contrast, no significant difference was observed in the effects of the virtual bolus on dose distribution for open fields between the two platforms (Δ = -22.0 cGy, P > 0.05). **Figure 6** presents the plan passing rates (γ[3%, 2 mm]) of the two plan groups. All plans achieved passing rates above 95%, meeting clinical treatment requirements. The Halcyon system demonstrated a slightly higher mean passing rate compared to the TrueBeam (Δ = 0.1%, P > 0.05), however, no statistically significant difference was observed between the two groups in terms of gamma passing rates.

## 4 Discussion

Breast cancer is the leading malignancy in females and increasingly affects younger individuals, urging the need for survival-prolonging strategies. This study compared dosimetric parameters between the novel dual-layer MLC ring-gantry system (Halcyon 3.0) and conventional C-arm accelerators (TrueBeam) for breast cancer treatment using VMAT technique. The results demonstrated that Halcyon 3.0 significantly reduces low-dose exposure to OARs while maintaining good target coverage and better homogeneity for clinical breast cancer planning.

The 5-year survival rate for breast cancer reaches approximately 90% (20). However, radiation-induced complications, including pulmonary injury, cardiotoxicity, secondary malignancies (e.g. contralateral breast cancer), spinal cord damage, hypothyroidism (in patients receiving supraclavicular nodal irradiation), and hepatic/gastric dysfunction, significantly threaten long-term survivorship and quality of life (21). This study evaluated dosimetric metrics for nearly all OARs relevant to breast radiotherapy, and found that Halcyon 3.0 demonstrated superior performance in breast cancer radiotherapy planning. To be specific, Halcyon 3.0 demonstrated MD reductions exceeding 50 cGy for each of the following parameters: ipsilateral lung Dmean, thyroid Dmean, and spinal cord Dmax, with statistically significant differences compared to TrueBeam (P< 0.05). For heart and LAD Dmean, the MD exceeded 100cGy dose reduction in Dmean, underscoring Halcyon’s enhanced cardiac sparing. Low-dose exposure (V5) was reduced by >8% for the heart, ipsilateral lung, and bilateral lungs. Halcyon also significantly decreased the V5 (P< 0.001).

We speculate that these differences may be due to three factors: 1) Halcyon’s Unique MLC Design: The dual-layer staggered MLC system minimizes radiation leakage (approximately 0.01%), directly contributing to reducing low-dose exposure (V5) and Dmean. Similarly, Li et al. found Halcyon demonstrated less MLC dose spillage compared to TrueBeam in early research (22); 2) Field Size Limitations of TrueBeam: The X-jaw travel limit of TrueBeam may partially constrain plan optimization, a critical geometry in breast radiotherapy; 3) Consistent Planning Parameters: TrueBeam plans were optimized using identical constraints and objectives as Halcyon, eliminating subjective optimization adjustments. However, Halcyon showed no advantage in high-dose regions (V20) and conformity (P > 0.05), though homogeneity was marginally better (P< 0.05). Virtual bolus integration to mitigate respiratory motion-induced underdosing revealed no inter-system differences (P > 0.05).

This study provides a comprehensive comparison of Halcyon 3.0 and TrueBeam for full VMAT breast planning, demonstrating Halcyon’s significant clinical dosimetric advantages of breast plan. Before this study, some investigations had been conducted on Halcyon systems. Biswal et al. reported the superior performance of Halcyon in reducing low-dose spillage compared to Novalis Tx for craniospinal irradiation VMAT planning (23). Li et al. demonstrated that Halcyon achieved better sparing of OARs in cervical cancer IMRT plans relative to Trilogy, an advantage attributed to Halcyon’s unique double-layer low-transmission MLC design and FFF beam delivery (24). Supporting these findings, Sun et al. observed enhanced OAR sparing in hippocampal-avoidance plans using triple-arc Halcyon VMAT compared to comparable TrueBeam and Trilogy plans (25). Their results all support our research inferences. Furthermore, we identified that this reduced dose leakage is particularly pronounced in low-dose regions (e.g., V5) in breast VMAT plans. Additionally, we expanded upon these insights by offering detailed dosimetric metrics specific to breast cancer VMAT planning.

Limitations include the modest sample size (n=30), which warrants validation in larger cohorts. Additionally, while our hypothesis that Halcyon’s reduced transmission benefits low-dose OARs sparing applies broadly to 3D-CRT and IMRT, this study focused solely on VMAT due to its widespread clinical adoption. Future work should incorporate 3D-CRT and IMRT comparisons for a holistic evaluation.

## 5 Conclusion

In a comprehensive evaluation, the Halcyon 3.0 platform demonstrated superior performance in generating higher-quality VMAT plans for breast cancer compared to TrueBeam. This superiority is particularly attributed to its enhanced low-dose leakage, which resulted in improved radiation doses of OARs highly susceptible to low-dose exposure, such as the heart, lungs, and contralateral breast. Additionally, Halcyon maintained optimal dose homogeneity and conformity within the target volumes.

## Data Availability

The raw data supporting the conclusions of this article will be made available by the authors, without undue reservation.
